# Mental Health Measurement in a Post Covid-19 World: Psychometric Properties and Invariance of the DASS-21 in Athletes and Non-athletes

**DOI:** 10.3389/fpsyg.2020.590559

**Published:** 2020-10-22

**Authors:** Robert S. Vaughan, Elizabeth J. Edwards, Tadhg E. MacIntyre

**Affiliations:** ^1^School of Education, Language and Psychology, York St John University, York, United Kingdom; ^2^School of Education, Faculty of Humanities and Social Sciences, The University of Queensland, Brisbane, QLD, Australia; ^3^Department of Physical Education and Sport Sciences, University of Limerick, Limerick, Ireland

**Keywords:** depression, anxiety, stress, athletes, mental health, psychometrics

## Abstract

Psychological science faces a call to action researching the implications of the corona virus disease 2019 (Covid-19) pandemic. Rapid reviews have reported that maintaining rigorous research standards is a priority for the field, such as ensuring reliable and valid measurement, when investigating people’s experience of Covid-19 (O’Connor et al., 2020). However, no research to date has validated a measure mental health symptomology for an athlete population. The current research addresses this gap by examining the internal consistency, factor structure, invariance, and convergent validity of the Depression Anxiety and Stress Scale (DASS-21; Lovibond and Lovibond, 1995) in two athlete samples. Participants completed the DASS-21 and sport-specific measures of mental health such as the Profile of Mood States – Depression subscale (POMS-D), Sport Anxiety Scale-2 (SAS-2), Athlete Burnout Questionnaire (ABQ), and Athlete Psychological Strain Questionnaire (APSQ). In sample one (*n* = 894), results of exploratory structural equation modeling indicated that a three-factor model provided good fit to the data, but a bifactor model provided better fit. Factor loadings indicated minimal misspecification and higher loadings on the general-factor. Invariance testing suggested equivalence across gender, athletic expertise, sport type, and injury status. Further, latent mean differences analyses indicated that females and injured athletes scored higher than male and non-injured athletes on all DASS-21 factors reporting higher mental health symptomology, those with more expertise scored higher on the general-factor and depression and those with less expertise scored higher on anxiety and stress, and no differences between team and individual athletes. In sample two (*n* = 589), the bifactor structure was replicated. Results largely supported the scales convergent validity with depression predicting POMS-D scores, whereas all three subscales predicted the SAS-2, ABQ, and APSQ scores. Internal consistency was acceptable in both samples. The current work provides initial support for use of the DASS-21 as an operationalisation of mental health symptomology in athletes. Theoretical and practical implications of these results are discussed.

## Introduction

Psychological science faces a call to action researching the implications of the corona virus disease 2019 (COVID-19) pandemic. Rapid reviews have reported that maintaining rigorous research standards is a priority for the field, such as ensuring reliable and valid measurement, when investigating peoples experience of COVID-19 (O’Connor et al., 2020). However, no research to date has validated a measure of mental health symptomology for an athlete population prior to or during the global lockdown. If the findings of research investigating the implications of COVID-19 for athletes are to be considered robust, and considering calls in the literature, then measurement accuracy and consistency needs to be established. That is, measurement assumptions are critical for the field moving forward ensuring confidence in findings which may inform policy, training, or treatment of athlete mental health.

Research has highlighted the psychological uniqueness of athlete populations (Reardon and Factor, 2010). For example, the athlete environment produces unique stressors as a result of high-pressure circumstances, constant mental effort, and experiences such as injury which may negatively impact mental health (Rice et al., 2016). It is plausible that these circumstances create difficulty when assessing or comparing athlete mental health with measures developed for the general population (Lebrun and Collins, 2017). Research has called for indices of mental health to be validated in athlete samples (Chiu et al., 2016). A popular measure of mental health is the Depression, Anxiety, and Stress Scale (DASS-21; Lovibond and Lovibond, 1995). Whilst research comparing athletes and non-athletes on the DASS-21 are scarce and inconsistent (Moghadasin et al., 2014; Bardhoshi et al., 2016; Demirel, 2016), no study has examined the invariance of mental health measures between elite, amateur and non-athletes. If effects are to be attributed to grouping factors rather than methodological reasons, the assumption of measurement invariance will be important (Marsh et al., 2011). Additionally, the psychometric properties of the DASS-21 are yet to be evaluated in a sport context, a gap addressed by the current work.

### Athlete Mental Health

O’Connor et al. (2020) suggest that COVID-19 will have severe and far reaching psychological consequences for society’s mental health. This effect is compounded considering the implications of COVID-19 for athletes such as restrictions on training, periods of isolation and cancelation of competition. Interest in athlete mental health has increased and is reflected in consensus statements regarding mental health identification in sport (Moesch et al., 2018; Henriksen et al., 2019). Much of this work highlights a link between the athlete environment and experiences of depression and anxiety. For example, serious injury causing early retirement and loss of identity, organizational-level pressures and occupational demands, public scrutiny of performance and person, have all been linked with mental health disorders (Foskett and Longstaff, 2018). Nonetheless, research examining athlete mental health is equivocal, with mental health prevalence reported at similar, below, and above general population rates (Rice et al., 2016; Lebrun et al., 2018). It is possible that differences may be attributed to limitations in measurement (Chiu et al., 2016), however, the most likely explanation for such discrepancies is that scales developed to measure constructs related to mental health in sport, are not direct tests of depression, anxiety, or stress (e.g., Sport Anxiety Scale-2, Smith et al., 2006) but contextualized operationalizations in relation to athlete performance (Smith et al., 2006). An evidence-based understanding of athlete mental health is lacking, therefore evaluation of appropriate scales is the first step in addressing this (Marsh et al., 2011, 2013). This is particularly important in a post COVID-19 world with researchers focusing on the mental health implications of the disease.

### Psychometric Properties

The DASS-21 (Lovibond and Lovibond, 1995) is a general measure of symptoms of depression, anxiety and stress (i.e., in the last 7 days). The scale was devised as a 42-item instrument discriminating symptoms of the non-diagnostic general depression-anxiety disorder or negative-affect into measures of depression, physical arousal, and psychological tension and agitation (Antony et al., 1998). The shortened 21-item scale, the focus of the current work, performs as well as the original and is considered the preferred version of the scale (Antony et al., 1998; Henry and Crawford, 2005). Although not yet validated with an athlete sample, much work outside of sport has supported the utility of the DASS-21 (e.g., Henry and Crawford, 2005), and psychometric examination consistently supports its internal consistency (Antony et al., 1998; Henry and Crawford, 2005; Osman et al., 2012; Wang et al., 2016; Shaw et al., 2017; Fox et al., 2018).

Whilst examination of the factor structure of the DASS-21 generally supports the three-factor model proposed by Lovibond and Lovibond (1995) across clinical, community, and non-clinical samples (Antony et al., 1998; Clara et al., 2001; Henry and Crawford, 2005), different countries, cultures, and languages (Mellor et al., 2015; Wang et al., 2016; Scholten et al., 2017; Kyriazos et al., 2018), those with and without obstructive sleep apnoea (Nanthakumar et al., 2017), or those with and without cancer diagnoses (Fox et al., 2018), there is debate regarding its optimal representation. That is, despite strong empirical support, the common underlying core of the DASS-21 supports the idea of a bifactor framework allowing use of total and subscale scores (i.e., tapping general negative-affect and specific factors simultaneously; Osman et al., 2012; Shaw et al., 2017; Kyriazos et al., 2018). Empirical work supports this notion. For example, Osman et al. (2012) reported that a bifactor model provided best fit to the data in two student samples. The factor loadings were acceptable, and the negative-affect or general-factor predicted more variance on a measure of mixed depression and anxiety. Shaw et al. (2017) proposed that the general-factor in a bifactor framework was a better representation of the DASS-21 items due to higher loadings and that the specific factors were non-invariant over adolescent ages groups.

Other work, however, contests a bifactor representation (Kyriazos et al., 2018). Kyriazos et al. (2018) examined the factor structure of the DASS-21 using exploratory (EFA) and confirmatory factor analysis (CFA), and exploratory structural equation modeling (ESEM) in a sample of 2,272 Greek adults. Despite reporting high indices of fit in a bifactor model, the authors suggested that the misspecification in the factor structure was unacceptable (e.g., several instances of cross and misloading items). However, it should be noted that some misspecification in the factor structure may be unavoidable in ESEM due to the multidimensional hierarchical framework specified by scales such as the DASS-21 (e.g., the moderate intercorrelations specified in order to obtain suitable internal consistency; Vaughan et al., 2018). Interestingly, Kyriazos et al. (2018) is the only example subjecting the DASS-21 to ESEM. The three-factor ESEM model reported better fit than the bifactor and final accepted three-factor CFA. Nonetheless, the authors rejected this model due to misspecification in the factor structure.

Research has advocated the benefits of ESEM in that it avoids the strict item specification requirements of CFA by allowing cross-loadings on non-intended factors, like in EFA, whilst providing robust indicators of model fit (Marsh et al., 2011, 2013). Research has integrated the bifactor framework into ESEM which may be especially relevant for the DASS-21 as it allows for estimation of both the hierarchical nature of the constructs being assessed (i.e., the co-existence of global and specific components within the same measurement model), and the degree of accuracy associated with the constructs’ indicators (i.e., how well items load on their target construct and the degree of overlap with non-target constructs; Henry and Crawford, 2005; Morin et al., 2016). A bifactor model specifies unique and common variance associated with the factors (Stenling et al., 2015). Regarding the DASS-21, bifactor ESEM would enable researchers to examine a general negative-affect factor and the specific depression, anxiety, and stress factors concurrently (Lovibond and Lovibond, 1995), and extends on the work of Kyriazos et al. (2018) who did not examine a bifactor ESEM – a gap this paper addresses.

### Measurement Invariance

Despite calls in the literature to validate measures of mental health with athletes, no study has examined the invariance of the DASS-21 in sport (Marsh et al., 2011; Chiu et al., 2016). To date, mixed support for invariance has been provided focusing largely on equivalence across countries. For example, Scholten et al. (2017) reported that the DASS-21 was invariant across the United States, Poland, Russia and the United Kingdom. Similarly, Mellor et al. (2015) reported invariance of the three-factor model across Australia, Chile, China and Malaysia. Invariance has also been supported over gender (Jafari et al., 2017; Lu et al., 2018). Other work reveals inconsistency over culture (e.g., Indonesia, Malaysia, Singapore, Sri Lanka, Taiwan, and Thailand) with several items omitted before acceptable model fit could be achieved (Oei et al., 2013). Additionally, measurement invariance across African-American/Black, Caucasian/White, Hispanic/Latino, and Asian could not be established due to large misspecification in the factor structures (Norton, 2007). It is possible that the use of idioms such as “wind down” in the item-set may create confusion outside non-native English speakers and thus discrepancy in the factor structure.

An implicit assumption of research when using the DASS-21 is that the items are interpreted the same across groups (Chen, 2007). Research in sport is scarce with findings reporting significant and non-significant differences between athlete groups (Moghadasin et al., 2014; Demirel, 2016). For example, Drew et al. (2017) indicated that female and injured athletes reported higher depression scores on the DASS-21, whereas Bardhoshi et al. (2016) reported significantly lower DASS-21 scores in senior games athletes compared to non-clinical normative data. One possible reason for this inconsistency is a lack of measurement invariance, that is, challenging the assumption that items operate equivalently across varying populations in respect of gender, age, and/or ability (Muthén and Muthén, 2017). Researchers are yet to examine this notion in sport (e.g., across gender, athletic expertise, sport type of injury status). Considering Marsh et al’s. (2011) suggestions not to use a scale across groups until invariance is confirmed, advancement of athlete mental health research will be dependent upon establishing whether differences between groups are attributable to theoretical or methodological reasons (Marsh et al., 2013). Validation of this assumption is vitally important as research increases investigating the impact of COVID-19 on athlete mental health.

### Convergent Validity

Research has most frequently compared the subscales of the DASS-21 against general measures of depression and anxiety such as the Beck Depression Inventory or Beck Anxiety Inventory (Beck and Steer, 1990; Beck et al., 1996; Gloster et al., 2008; Wang et al., 2016). The DASS-21 correlates positively with these measures and other related measures such as the negative-affect subscale of the PANAS (Watson et al., 1988). Support for the depression and anxiety subscales are consistent, however, support for the stress subscale is lacking. For example, Gloster et al. (2008) reported a positive relationship between the stress subscale and the negative-affect subscale of the PANAS, the Beck Depression Inventory, and the Beck Anxiety Inventory. Whilst it appears the stress subscale is tapping negative-affect and other measures of mental health, the lack of a stress specific measure creates difficulty in ascertaining whether the scale is measuring general disorders or specific stress symptoms. Similarly, Andreou et al. (2011) reported positive correlations between the DASS-21 subscales and the Perceived Stress Subscale (Cohen et al., 1983). Together, this work provides a foundation for assessing the DASS-21’s convergent validity and whether the scale is transferable to a sport context capable of capturing elements of depression, anxiety and stress in athletes. Although well-established outside of sport, little work has examined the relationship between the Profile of Moods State – Depression subscale (i.e., depression; Grove and Prapavessis, 1992), the Sport Anxiety Scale-2 (i.e., anxiety; Smith et al., 2006), and the DASS-21.

Research has suggested that examination of the DASS-21’s convergent validity is lacking despite its critical importance (Lee, 2019). For example, convergence analysis between the DASS-21 Stress subscale and the Athlete Burnout Questionnaire (Raedeke and Smith, 2001) would demonstrate if this subscale is able to capture the athlete-specific concept of burnout. Burnout in sport manifests as a response to chronic stress produced by the demanding nature of the athlete environment and is considered incomparable to burnout experienced in other contexts (e.g., occupational settings; Gustafsson et al., 2018). De Francisco et al. (2016) reported a positive relationship between the stress subscale and athlete burnout in a structural equation modeling framework. However, this research failed to control for the overlap between the depression and anxiety subscales of the DASS-21.

Synonymous with mental health is the concept of psychological strain, a combination of perceived stress and difficulty coping, that has recently been operationalised in sport (Rice et al., 2019). Whilst research posits a positive relationship between the constructs outside of sport (Leung et al., 2009), it is unclear whether the DASS-21 is able to account for cross-domain differences in the conceptualisation of psychological strain, depression, anxiety or stress.

### The Present Study

The aim of the present study was to determine the utility of the DASS-21 as an appropriate operationalisation of mental health in sport by assessing the psychometric properties of the DASS-21 across two samples of athletes. Establishing internal consistency, factorial validity, measurement invariance, and convergent validity in sport will ensure methodological rigor for future work investigating the implications of COVID-19 (O’Connor et al., 2020). In sample one, we examined the factor structure using ESEM comparing the original three-factor model, a single-factor model, and a bifactor model. Second, we examined the invariance of the scale across gender, athletic expertise, sport type, and injury status. Third, we tested group differences and internal consistency. Next, in sample two, we replicated the factor structure and inspected the convergent validity of the scale against sport-specific measures of stress, anxiety, depression and psychological strain providing a two-stage analysis of the DASS-21’s psychometric properties. Although, little prior work exists with athletes we predicted that a bifactor model would provide the best fit to the data. We also hypothesized that the DASS-21 would be invariant and that scores would differ across gender, athletic expertise, sport type, and injury status groups. Finally, we expect the DASS-21 subscales to correlate significantly with measures of athlete burnout, competitive anxiety, depressive feelings, and psychological strain.

## Materials and Methods

### Participants

Sample one comprised 894 participants (54.14% female) aged 17–57 years (*M*^*age*^ = 32.66, *SD* = 12.14). Participants were elite (*n* = 252), amateur (*n* = 309) and non-athletes (*n* = 333) from various team (*n* = 329) and individual (*n* = 232) sports (e.g., athletics, rugby, soccer, tennis, and volleyball) with (*n* = 218) and without (*n* = 343) injury. Classification of athlete status was based on Swann et al. (2015) criteria similar to previous psychometric work with athletes (Vaughan et al., 2018). Non-athletes were those who did not compete in any sport and failed to score on Swann et al. (2015) criteria.

Sample two consisted of 589 athletes (57.21% male) aged 18–41 years (*M*^*age*^ = 23.54, *SD* = 9.38) with an average of 8.82 years playing experience. All athletes competed regularly in a range of sports at the time of participation (e.g., soccer and tennis).

Myers et al. (2017) recommend the use of Monte Carlo simulation for estimation of sample size in structural equation modeling, however, no guidelines exist for parameter estimation in ESEM. Therefore, applying CFA estimations with no missing data, standard error biases that do not exceed 10%, and coverage of confidence intervals set at 95%, sufficient power (80%) could be achieved with a sample size of 580 (Muthén and Muthén, 2017).

### Materials

The DASS-21 (Lovibond and Lovibond, 1995) is a 21-item self-report questionnaire which assess recent experiences of stress (e.g., “I found it hard to wind down”), anxiety (e.g., “I felt close to panic”), and depression (e.g., “I felt that I had nothing to look forward to”). Each 7-item subscale is rated on a 4-point Likert scale ranging from 0 (Did not apply to me at all) to 3 (Applied to me very much). Higher scores represent greater symptomology.

The Sport Anxiety Scale-2 (SAS-2; Smith et al., 2006) is a 15-item self-report questionnaire of competitive anxiety. Responses are rated on a four-point Likert scale ranging from 1 (Not at all) to 4 (Very much) assessing somatic anxiety (e.g., “My stomach feels upset”), worry (e.g., “I worry that I will let others down”) and concentration disruption (e.g., “I lose focus on the game”), with five items each. Higher scores indicated increased anxiety. Previous research has provided psychometric support (e.g., measurement invariance across gender and sport type) for the scale (Ramis et al., 2015). Internal consistency was supported in the current work (Ω = 0.74–0.78).

The Abbreviated Profile of Mood States Questionnaire (POMS-D; Grove and Prapavessis, 1992) is a 40-item self-report measure which assess seven different mood states (i.e., tension, anger, fatigue, depression, esteem-related affect, vigor, and confusion). Responses are provided on a five-point Likert scale ranging from 0 (Not at all) to 4 (Extremely) to an adjective checklist (e.g., tense, angry, sad, active, restless, proud). In the present research, only the depression subscale was used. Previous research has supported the psychometrics of the scale and the invariance of the POMS when used with athletes (Andrade and Rodriguez, 2018).

The Athlete Burnout Questionnaire (ABQ; Raedeke and Smith, 2001) is a 15-item self-report questionnaire of athlete burnout. Participants respond on a five-point Likert scale ranging from 1 (almost never) to 5 (almost always) assessing reduced sense of accomplishment (e.g., “I’m accomplishing many worthwhile things in [sport]”), emotional/physical exhaustion (e.g., “I feel physically worn out from [sport]”), and sport devaluation (e.g., “I’m not into [sport] like I used to be”). Previous research has supported the ABQs factor structure and internal consistency (Gerber et al., 2018). Internal consistency was supported in the current work (Ω = 0.73–0.79).

The Athlete Psychological Strain Questionnaire (APSQ; Rice et al., 2019) is a 12-item measure of sport-specific psychological strain. Responses are provided on a five-point Likert scale ranging from 1 (none of the time) to 5 (all of the time) assessing self-regulation (e.g., “I was less motivated”), performance (e.g., “I found training more stressful”), and external coping (e.g., “I took unusual risks off-field”). Higher scores indicate greater psychological strain. Rice et al. (2019) provided support for factorial, convergent, and divergent validity in APSQ’s development. Internal consistency was supported in the current work (Ω = 0.71–0.73).

### Procedure

Ethical approval was granted from a university ethics committee. Data were collected at designated laboratories at a university psychology department or during athletes training. Participants were briefed prior to data collection, informed of their ethical rights, and provided informed consent. Participants provided demographic information (e.g., age and sex), athlete status (e.g., participated in sport or not, which sport, how long, what level of competition, and highest level of success), and injury status. Participants then completed the DASS-21, SAS-2, POMS-D, ABQ, and APSQ. Data were first entered onto SPSSv24 for preliminary analyses and then Mplus 7.4 for model testing (Muthén and Muthén, 2017).

### Design and Data Screening

We adopted a cross-sectional design with purposive sampling. A small amount of data were missing (1.4%). Following recommendations (Tabachnick and Fidell, 2007), we used ipstatized estimation of relevant cases. Multivariate skewness (33.15, *p* > 0.05) and kurtosis (56.97, *p* > 0.05) coefficients indicated no departure from normality (Muthén and Muthén, 2017).

### Analytic Strategy

First, we calculated means, standard deviations, and internal consistency (omega; Dunn et al., 2014) for all variables (Table 1). Next, we tested a one and three-factor model using ESEM and then a bifactor-ESEM with latent means analysis (see Gucciardi and Zyphur, 2016). Then, we assessed measurement invariance across gender, athletic expertise, sport type, and injury status on the best fitting model (Muthén and Muthén, 2017). Measurement invariance was tested between the configural model (i.e., the same pattern of factors and loadings across groups), metric model (i.e., invariant loadings), and scalar model (i.e., invariant factor loadings and intercepts). For these analyses, we used the robust maximum likelihood estimator (Muthén and Muthén, 2017). The robust maximum likelihood estimator can handle instances of missing data, non-normality, categorical variables when there are at least five response categories, and is particularly suited to bifactor interpretations compared to other estimators (Stenling et al., 2015). We also assessed latent means differences between groups after measurement invariance is at least partially established (Vandenberg and Lance, 2000).

**TABLE 1:** DASS-21 descriptive statistics across gender, athletic expertise, sport type, and injury status.

	**Overall**	**Gender**			**Athletic expertise**				**Sport type**			**Injury status**			
		**Male**	**Female**		**Non**	**Amateur**	**Elite**		**Team**	**Individual**		**Non-injured**	**Injured**		

**Scale**	***M* (*SD*)**	***M* (*SD*)**		***d***	***M* (*SD*)**			***d***	***M* (*SD*)**		***d***	***M* (*SD*)**		***d***	**Ω**
Total score	6.92 (2.71)	4.87 (1.62)	8.93 (2.70)	0.04**	4.88 (1.61)	6.91 (1.64)	7.95 (1.67)	0.06**	6.91 (2.68)	6.94 (2.65)	0.01	4.86 (.69)	8.96 (.74)	0.10**	0.84
Depression	5.54 (1.55)	4.59 (1.51)	6.52 (1.45)	0.03*	4.51 (1.57)	5.56 (1.51)	6.61 (.48)	0.05**	5.52 (1.59)	5.57 (1.61)	0.01	3.51 (1.52)	7.59 (1.54)	0.06**	0.82
Anxiety	4.43 (1.48)	3.41 (1.49)	5.48 (1.39)	0.04**	5.49 (1.47)	4.41 (1.45)	3.36 (1.48)	0.03*	4.44 (1.41)	4.45 (1.49)	0.01	3.41 (1.47)	5.49 (1.42)	0.05**	0.79
Stress	7.86 (1.60)	6.82 (2.55)	8.89 (2.62)	0.03*	9.89 (2.61)	7.84 (2.52)	5.79 (1.56)	0.04**	7.88 (1.64)	7.85 (1.61)	0.01	5.81 (2.58)	9.91 (1.62)	0.05**	0.81

N = 894. *p < 9.05; **p < 0.01.

As an hypothesized model exists regarding the factor structure of the DASS-21, an oblique target and oblique-bifactor target rotation were used to estimate how the 21-items and latent factors of the DASS-21 were interrelated for the ESEM and bifactor-ESEM, respectively. An epsilon value of 0.50 was adopted to enable as many items as possible to be optimally identified within one component while minimizing the potential number of doublets (Comrey and Lee, 1992). To evaluate model fit, we examined the χ^2^ statistic, comparative fit index (CFI), Tucker–Lewis Index (TLI), Root Mean Square Error of Approximation (RMSEA) with 95% Confidence Intervals (CI), and Standardized Root Mean Square Residual (SRMR) using the following criteria: *CFI* > 0.90, *TLI* > 0.90, *RMSEA* < 0.06, *SRMR* < 0.06 (Marsh et al., 2004).

In order to select the most parsimonious model, the Bayesian Information Criterion (BIC) and Akaike’s Information Criterion (AIC) were used to compare models. The AIC and BIC assign a greater penalty to model complexity and therefore have a better propensity to select more efficient models. In addition, a change of less than 0.01 in the CFI and 0.015 in the RMSEA support an invariant model in relation to the previous model (Chen, 2007). Due to the exploratory nature of ESEM, standardized solutions were examined to evaluate the significance and strength of parameter estimates. The following criteria were used to evaluate the standardized factor loadings (>0.71 = excellent, >0.63 = very good, >0.55 = good, >0.45 = fair, >0.32 = poor; Comrey and Lee, 1992).

Finally, we used multiple linear regression with the DASS-21 factors as predictors to examine their influence on the sport-specific measures as outcome variables. Positive associations with similar concepts support convergent validity (Rice et al., 2019).

## Results

### Preliminary Analyses and Reliability

Descriptive statistics were calculated for the total and subscale scores from sample one. Omega values were satisfactory (Ω = 0.79–0.84) for a composite and subscale scores (see Table 1).

### Factor Structure

The one-factor model did not provide a good fit to the data. The three-factor model provided improved and acceptable fit. However, a bifactor model provided the best fit to the data (χ^2^ [194] = 1325.927, *p* < 0.05; RMSEA = 0.047 [0.044–0.051]; SRMR = 0.043; TLI = 0.928; CFI = 0.945; AIC = 94784.621; BIC = 97153.232). Model comparison revealed best fit with the bifactor model with lower AIC and BIC values compared to both one and three-factor representations (see Table 2).

**TABLE 2:** Model fit indices for sample one and two.

**Sample**	**Model**	**χ^2^**	***df***	**RMSEA (90% CI)**	**SRMR**	**TLI**	**CFI**	**AIC**	**BIC**
One (*N* = 894)	ESEM (one factor)	2145.654	241	0.069 (0.064–0.071)	0.063	0.821	0.847	95325.854	98627.523
	ESEM (three factor)	1846.631	215	0.058 (0.054–0.060)	0.054	0.905	0.917	95174.681	97853.143
	Bifactor-ESEM	1325.927	194	0.047 (0.044–0.051)	0.043	0.928	0.945	94784.621	97153.232
	Gender configural	1897.582	425	0.058 (0.055–0.060)	0.054	0.912	0.924	94812.628	97329.674
	Gender metric	1748.603	512	0.056 (0.053–0.059)	0.052	0.909	0.919	94531.654	97214.971
	Gender scalar	1658.935	547	0.054 (0.051–0.056)	0.051	0.902	0.913	94413.369	97028.147
	Expertise configural	1987.267	628	0.058 (0.055–0.061)	0.057	0.917	0.924	93528.289	96107.927
	Expertise metric	2517.057	738	0.059 (0.056–0.062)	0.059	0.910	0.918	93824.317	96389.829
	Expertise scalar	2923.742	791	0.060 (0.057–0.063)	0.058	0.905	0.913	94153.974	96745.282
	Type configural	1954.923	451	0.057 (0.055–0.060)	0.053	0.913	0.922	94233.748	97592.382
	Type metric	1867.328	518	0.055 (0.052–0.058)	0.052	0.909	0.916	94117.825	97381.537
	Type scalar	1726.628	554	0.052 (0.050–0.055)	0.051	0.901	0.907	93924.743	97106.581
	Injury configural	2097.965	508	0.057 (0.054–0.059)	0.055	0.917	0.928	94344.627	97157.319
	Injury metric	2031.836	574	0.056 (0.054–0.059)	0.052	0.912	0.917	94187.323	96543.253
	Injury scalar	1974.434	632	0.058 (0.055–0.060)	0.051	0.904	0.911	93821.652	96204.826
Two (*N* = 589)	ESEM (three factor)	1745.358	215	0.056 (0.051–0.059)	0.055	0.914	0.926	95028.921	97641.638
	Bifactor-ESEM	1307.139	194	0.046 (0.046–0.052)	0.042	0.932	0.949	94121.347	97008.391

*χ^2^ = Chi-Square, RMSEA, Root Mean Square Error of Approximation; CI, Confidence Interval; SRMR, Standardized Root Mean Residual; TLI, Tucker Lewis Index; CFI, comparative fit index; AIC, Akaike Information Criterion; BIC, Bayesian Information Criterion.*

The standardized factor loadings indicated higher loadings for the general-factor than for the specific factors with a range of fair to excellent loadings (e.g., 0.45–0.86). For each factor, six of the highest loading items were located on the general-factor except for items 16, 2, and 14 for the depression, anxiety and stress, respectively. Higher factor loadings in the general as opposed to specific factors supports a bifactor representation highlighting the shared conceptual underpinning of the DASS-21 (Marsh et al., 2004). Whilst some significant cross-loadings were found (e.g., items 3, 13, 17, 4, 9, 19, 6, 11, and 14), none of these reached the predetermined cut-off (e.g., >0.32; Comrey and Lee, 1992). Positive correlations (*r* = 0.52–0.80) were found between latent factors (see Table 3).

**TABLE 3:** Parameter estimates and latent factor correlations for bifactor model.

	**Sample one (*N* = 894)**				**Sample two (*N* = 589)**			
**Item**	**General**	**Factor 1**	**Factor 2**	**Factor 3**	**General**	**Factor 1**	**Factor 2**	**Factor 3**
**Depression**								
3. I couldn’t seem to experience any positive feeling at all	0.635**	0.592**	0.203*	0.154	0.588**	0.438**	0.141	0.016
5. I found it difficult to work up the initiative to do things	0.762**	0.587**	0.115	0.178	0.417**	0.734**	0.108	0.047
10. I felt that I had nothing to look forward to	0.738**	0.571**	0.182	0.192	0.716**	0.564**	0.084	0.095
13. I felt down-hearted and blue	0.529**	0.491**	0.221*	0.144	0.513**	0.457**	0.027	0.104
16. I was unable to become enthusiastic about anything	0.407**	0.753**	0.134	0.117	0.421**	0.714**	0.039	0.029
17. I felt I wasn’t worth much as a person	0.866**	0.581**	0.216*	0.191	0.748**	0.627**	0.326**	0.007
21. I felt that life was meaningless	0.467**	0.421**	0.156	0.147	0.492**	0.449**	0.109	0.061
**Anxiety**								
2. I was aware of dryness of my mouth	0.395**	0.142	0.799**	0.187	0.387**	0.055	0.781**	0.129
4. I experienced breathing difficulty (e.g., excessively rapid breathing, breathlessness in the absence of physical exertion)	0.554**	0.217*	0.451**	0.105	0.729**	0.031	0.477**	0.131
7. I experienced trembling (e.g., in the hands)	0.836**	0.157	0.468**	0.114	0.691**	0.112	0.492**	0.321**
9. I was worried about situations in which I might panic and make a fool of myself	0.789**	0.216*	0.491**	0.141	0.407**	0.131	0.768**	0.094
15. I felt I was close to panic	0.637**	0.176	0.573**	0.186	0.583**	0.008	0.524**	0.092
19. I was aware of the action of my heart in the absence of physical exertion (e.g., sense of heart rate increase, heart missing a beat)	0.731**	0.234*	0.474**	0.193	0.631**	0.004	0.451**	0.004
20. I felt scared without any good reason	0.622**	0.128	0.582**	0.139	0.604**	0.067	0.575**	0.055
**Stress**								
1. I found it hard to wind down	0.655**	0.151	0.163	0.508**	0.587**	0.008	0.117	0.523**
6. I tended to over-react to situations	0.851**	0.127	0.238*	0.487**	0.381**	0.044	0.154	0.806**
8. I felt that I was using a lot of nervous energy	0.634**	0.128	0.127	0.492**	0.639**	0.151	0.329**	0.568**
11. I found myself getting agitated	0.722**	0.215*	0.121	0.495**	0.722**	0.118	0.008	0.463**
12. I found it difficult to relax	0.836**	0.142	0.133	0.528**	0.546**	0.136	0.087	0.507**
14. I was intolerant of anything that kept me from getting on with what I was doing	0.402**	0.169	0.227*	0.852**	0.382**	0.071	0.112	0.794**
18. I felt that I was rather touchy	0.766**	0.111	0.172	0.464**	0.654**	0.035	0.061	0.448**
**Latent Correlations**								
General		0.780**	0.752**	0.803**		0.713**	0.648**	0.761**
Depression			0.518**	0.585**			0.487**	507**
Anxiety				0.570**				0.494**

Values in bold indicate highest loading on that factor. Values underlined are interpreted as a factor. General, General Factor; Factor 1, Depression; Factor 2, Anxiety; Factor 3, Stress; See Lovibond and Lovibond (1995) for DASS-21 publication manual. *p < 0.05; **p < 0.01.

### Invariance Testing

Next, we tested invariance of the bifactor model across groups by comparing the configural (e.g., all parameters allowed to be unequal across groups) against the metric (e.g., holding loadings equal across groups) model which is a test of weak invariance followed by a test of strong invariance comparing the metric against the scalar (e.g., constraining factor loadings and intercepts across groups) model (see Table 2).

Invariance testing indicated equivalence across males and females. Subsequent increases in model constraint across gender revealed no significant difference between the configural and metric (Δ*χ*^2^ [87] = 148.979, *p* > 0.05), and metric and scalar (Δ*χ*^2^ [35] = 89.668, *p* > 0.05) models. Change in fit were within range of invariance and indicated acceptable fit to the data (Marsh et al., 2004; Chen, 2007). Also, the AIC and BIC were lowest for the configural model. Findings indicate that the DASS-21 remains invariant with each successive parameter restraint supporting the utility of the scale across gender.

Invariance testing indicated equivalence across elite, amateur and non-athletes. Subsequent increases in model constraint across athletic expertise revealed no significant difference between the configural and metric (Δ*χ*^2^ [110] = 529.791, *p* > 0.05), and metric and scalar (Δ*χ*^2^ [53] = 406.685, *p* > 0.05) models. Change in fit were within range of invariance and indicated acceptable fit to the data (Marsh et al., 2004; Chen, 2007). Also, the AIC and BIC were lowest for the configural model. Findings indicate that the DASS-21 remains invariant with each successive parameter restraint supporting the utility of the scale across athletic expertise.

Invariance testing indicated equivalence across team and individual athletes. Subsequent increases in model constraint across sport type revealed no significant difference between the configural and metric (Δ*χ*^2^ [67] = 87.595, *p* > 0.05), and metric and scalar (Δ*χ*^2^ [36] = 140.700, *p* > 0.05) models. Change in fit were within range of invariance and indicated acceptable fit to the data (Marsh et al., 2004; Chen, 2007). Also, the AIC and BIC were lowest for the configural model. Findings indicate that the DASS-21 remains invariant with each successive parameter restraint supporting the utility of the scale across sport type.

Invariance testing indicated equivalence across non-injured and injured athletes. Subsequent increases in model constraint across injury status revealed no significant difference between the configural and metric (Δ*χ*^2^ [66] = 66.129, *p* > 0.05), and metric and scalar (Δ*χ*^2^ [58] = 57.402, *p* > 0.05) models. Change in fit were within range of invariance and indicated acceptable fit to the data (Marsh et al., 2004; Chen, 2007). Also, the AIC and BIC were lowest for the configural model. Findings indicate that the DASS-21 remains invariant with each successive parameter restraint supporting the utility of the scale across injury status.

### Parameter Estimates for Invariance Measurement Models

Comparison of factor loadings support invariance with minimal misspecification supporting Lovibond and Lovibond’s (1995) model (see Supplementary Material). Inspection of the factor loadings and residual variances across gender indicate support for the hypothesized model (i.e., two cross-loading items per subscale). Similar levels of misspecification were found between genders with slightly larger loadings for females. Inspection of the factor loadings and residual variances across athletic expertise revealed a similar degree of misspecification (i.e., at least three cross-loading items per factor). The least amount of misspecification was noted for non-athletes, however, larger loadings were found for elite athletes. Factor loadings across sport type revealed three significant cross-loading in both team and individual athlete groups. Factor loadings were highest for team athletes. Comparison of the factor matrices across non-injured and injured athletes revealed the most amount of misspecification with at least four cross-loadings per factor. The smallest loadings and least amount of misspecification was noted for non-injured athletes. In most instances factor loadings were higher on the general-factor compared to specific factors (i.e., at least 4 items loaded highest on the general-factor across all models). The latent factor correlations indicated similar patterns across groups with positive relationships observed between the factors (see Supplementary Material).

### Latent Mean Differences

As invariance estimates were reasonable based on the recommendations of Chen (2007) we proceeded to test latent mean differences (see Table 1). Results indicated small significant differences in Cohen’s *d* between groups, with females and injured athlete scoring higher than males and non-injured athletes on all factors indicating more mental health symptomology, those with more expertise scored higher on the general-factor and depression whereas those with less expertise scored higher on anxiety and stress, and no differences between team and individual athletes.

### Replication of Factor Structure

In order to examine consistency of the model we tested the previously supported three and bifactor models in sample two (see Table 2). Similar to sample one, model fit was acceptable in both instances with better fit observed in the bifactor model (χ^2^ [194] = 1307.139, *p* < 0.05; RMSEA = 0.046 [0.042–0.052]; SRMR = 0.042; TLI = 0.932; CFI = 0.949; AIC = 94121.347; BIC = 97008.391). This model also indicated acceptable levels of internal consistency (Ω = 0.75–0.81). The factor loadings followed a similar pattern showing agreement with Lovibond and Lovibond’s (1995) model with a range of fair to excellent loadings (0.45–0.81). However, the factor structure did differ. In sample two, five items for each subscale loaded onto the general-factor above their target factor. Additionally, misspecification was found with one significant cross-loading in each factor. Whilst each instance of misloading was significant and just above the 0.32 cut-off (Comrey and Lee, 1992), higher factor loadings were found on the target and higher again on the general-factor. Again, positive correlations were observed between the factors (*r* = 0.49–0.76).

### Convergent Validity

Multiple regression models were constructed to examine how the DASS-21 subscales predicted measures of depression, competitive anxiety, athlete burnout, and athlete psychological strain (see Table 4). Results indicated that the DASS-21 predicted 38% of the POMS-D variance with depression the largest and only significant predictor supporting the convergent validity. Results indicated that the DASS-21 predicted between 12 and 19% of the SAS-2 variance. The largest predictor was anxiety supporting convergent validity, however, depression and stress also positively predicted the variance questioning the scales convergent validity. Results indicated that the DASS-21 predicted between 9 and 21% of the ABQ variance. Whilst in most cases stress was the largest predictor supporting convergent validity, depression was the largest predictor of the reduced accomplishment and sports devaluation subscales. Moreover, depression and anxiety positively predicted the ABQ variance questioning its convergent validity. Results indicated that the DASS-21 predicted between 11 and 27% of the APSQ variance. Specifically, depression was the largest predictor of self-regulation, anxiety was the largest predictor of performance, depression and anxiety equally predicted external coping, and depression was the largest predictor of athlete psychological strain. It should be noted that depression, anxiety and stress were positively related to each APSQ score therefore supporting scale’s convergent validity.

**TABLE 4:** Summary of multiple linear regressions.

**Model**	**POMS-D**		**SAS-2-S**		**SAS-2-W**		**SAS-2-CD**		**SAS-2-TA**		**ABQ-E**		**ABQ-RA**		**ABQ-SD**		**ABQ-B**		**APSQ-S**		**APSQ-P**		**APSQ-E**		**APSQ-T**	
*R*^2^	0.38**		0.12**		0.19**		0.16**		0.15**		0.09**		0.21**		0.18**		0.16**		0.11**		16**		0.19**		0.27**	

	**β**	**Part**	**β**	**Part**	**β**	**Part**	**β**	**Part**	**β**	**Part**	**β**	**Part**	**β**	**Part**	**β**	**Part**	**β**	**Part**	**β**	**Part**	**β**	**Part**	**β**	**Part**	**β**	**Part**
Depression	0.28**	0.41	0.11*	0.07	0.19**	0.13	0.11*	0.11	0.18**	0.13	0.11*	0.09	0.27**	0.19	0.23**	0.19	0.21**	0.17	0.17*	0.14	0.15*	0.11	0.16*	0.12	0.24**	0.19
Anxiety	0.06	0.04	0.16*	0.12	0.21**	0.15	0.16**	0.08	0.21**	0.17	0.10*	0.09	0.12*	0.09	0.18**	0.05	0.12*	0.09	0.13*	0.10	0.17*	0.14	0.16*	0.12	0.18**	0.14
Stress	–0.00	–0.00	0.14*	0.11	0.18**	0.12	0.09*	0.04	0.17**	0.11	0.13*	0.10	0.16**	0.14	0.20**	0.06	0.26**	0.19	11*	0.08	0.14*	0.08	0.15*	0.10	0.21**	0.17

N = 589. DASS, Depression Anxiety Stress Scale; POMS, Profile of Mood States; SAS-2, Sports Anxiety Scale; ABQ, Athlete Burnout Questionnaire; APSQ, Athlete Psychological Strain Questionnaire; DASS-S, DASS-21 Stress; DASS-A, DASS-21 Anxiety; DASS-D, DASS-21 Depression; POMS-D, Abbreviated POMS Depression; SAS-2-S, SAS Somatic; SAS-2-W, SAS Worry; SAS-2-CD, SAS concentration disruption; SAS-2-TA, SAS Trait Anxiety; ABQ-E, ABQ Physical/Emotional Exhaustion; ABQ-RA, ABQ Reduced Sense of Accomplishment; ABQ-SD, ABQ Sport Devaluation; ABQ-B, ABQ Burnout; APSQ-S, APSQ self-regulation; APSQ-P, APSQ performance; APSQ-E, APSQ External coping; APSQ-T, APSQ Total; β, standardized beta coefficient; Part, semi-partial correlations. *p < 0.05; **p < 0.01.

To further test the bifactor model, regression models indicated that the general factor significantly predicted between 8 and 36% of the POMS-D (i.e., depression), SAS-2 (i.e., somatic, worry, concentration disruption, trait anxiety), ABQ (i.e., exhaustion, reduced accomplishment, sport devaluation, and athlete burnout), APSQ (i.e., self-regulation, performance, external coping, and psychological strain) variance.

## Discussion

The aim of the present study was to assess the psychometric properties of the DASS-21 and provide evidence for its utility as a measure of athlete mental health in light of the COVID-19 pandemic. We investigated the fit of a one, three and bifactor representation. We explored measurement invariance of the DASS-21 across gender, athletic expertise, sport type and injury status and assessed the latent mean differences across these groupings. Along with replication, we also examined the convergent validity of the DASS-21 against sport specific measures of mental health. Results supported the psychometrics of the DASS-21 in a sport context thus providing researchers with a reliable and valid operationalisation of mental health moving forward (O’Connor et al., 2020). A bifactor representation provided the best fit to the data and was invariant across gender, athletic expertise, sport type and injury status. Whilst some misspecification was reported, these were below predetermined cut-offs. We also found small differences on the DASS-21 with females scoring higher than males, injured athletes scoring higher than non-injured athletes, those with more expertise scoring higher on the general factor and depression and those with less expertise scoring higher on anxiety and stress, and no differences between team and individual athletes (Moghadasin et al., 2014; Bardhoshi et al., 2016; Demirel, 2016). Also, we replicated the bifactor structure in an additional sample further demonstrating the scales utility. Factor matrices followed a similar pattern across samples and internal consistency was supported in both samples.

The differences reported corroborate previous research suggesting that athletes, particularly female and injured, will experience greater depression, anxiety, and stress symptomology (Demirel, 2016; Rice et al., 2016; Drew et al., 2017; Lebrun and Collins, 2017). The current research is the first to adopt an accepted framework of athletic expertise (Swann et al., 2015), revealing that those with more expertise experience greater negative-affect and depression whereas those with less expertise experience greater anxiety and stress. The differences reported on the negative-affect factor align with previous work suggesting that elite level sport may have negative impact on mental health (Moesch et al., 2018; Henriksen et al., 2019). Results indicated high internal consistency at the general and subscale level supporting previous research (Osman et al., 2012; Shaw et al., 2017; Fox et al., 2018).

### Psychometric Properties

The bifactor structure of the DASS-21 supports previous work (Osman et al., 2012; Shaw et al., 2017; Kyriazos et al., 2018), but also integrates Lovibond and Lovibond’s (1995) original conceptualisation as it suggests the co-existence of the negative-affect and depression, anxiety and stress components within the same model. Explanations for these findings are housed in the existence of negative-affect, depression, anxiety and stress in the DASS-21. First, the lack of fit in the one-factor model suggests that the individual factors capture variance not associated with a general negative-affect factor. Although, acceptable fit was observed in the three-factor model, the addition of a general-factor in the bifactor model improved fit. Third, the higher loadings on the negative-affect factor compared to their intended factor suggests that the items are not pure measures of each factor. It is possible that although depression, anxiety and stress may manifest uniquely, their shared underlying conceptual core means that overlap is unavoidable (Henry and Crawford, 2005). This is common in many aggregate scales where attempts to increase internal consistency high inter-item correlation is a by-product (Vaughan et al., 2018).

Regarding the structure of the specific factors, although generally acceptable in sample one, some misspecification remained in sample two, suggesting some items may be problematic. Specifically, item-17 of depression cross-loaded onto anxiety (i.e., “I felt I wasn’t worth much as a person”), item-7 of anxiety cross-loaded onto stress (i.e., “I experienced trembling e.g., in the hands”), and item-8 of stress cross-loaded onto anxiety (i.e., “I felt that I was using a lot of nervous energy”). Although no common theme appears between the items it is possible that athletes may place specific value on self-worth, nervousness and agitation resulting in misspecification across the factor structure (Grove and Prapavessis, 1992).

Higher factor loadings were generally observed in the general factor except for item-16 (e.g., “I was unable to become enthusiastic about anything”) of depression, item-2 (e.g., “I was aware of dryness of my mouth”) of anxiety, and item-14 (e.g., “I was intolerant of anything that kept me from getting on with what I was doing”) of stress in sample one, and items 16 and 5 (e.g., “I found it difficult to work up the initiative to do things”) of depression, items 2 and 9 (e.g., “I was worried about situations in which I might panic and make a fool of myself”) of anxiety, and items 14 and 6 (e.g., “I tended to over-react to situations”) of stress in sample two. In both samples’ items 16, 2, and 14 loaded higher on their intended factors (depression, anxiety, and stress, respectively) to a good level (Comrey and Lee, 1992). Previous research has suggested that item 2 may be problematic (Norton, 2007), however, this was not the case in the current data. It may be that dryness in the mouth was a common symptom for those who participate in sport. Interestingly, the other target loading items are all themed around motivation which is particularly important for those involved with sport and has links with mental health (Ng et al., 2012).

Moreover, although advantageous in ESEM, the identification of non-target rotations, may indicate redundancy or oversimplification in shortened scales such as the DASS-21 (Morin et al., 2016). For example, according to Lovibond and Lovibond (1995) the DASS-21 captures elements of depression (e.g., low self-esteem, dysphoria, lack of interest, displeasure, sense of hopelessness, devaluation of life, self-deprecation, low positive affect, lack of interest or involvement, anhedonia, and inertia), anxiety (e.g., autonomic arousal, fearfulness, skeletal musculature affects, situational anxiety, and subjective experience of anxiety and panic), and stress (e.g., lack of relaxation, nervous arousal, agitation, ease of becoming upset, irritability, negative-affect and impatience) which will inevitably result in factor overlap (Osman et al., 2012; Shaw et al., 2017).

### Measurement Invariance

Overall, the DASS-21 was fully invariant over gender, athletic expertise, sport type and injury status as indicated by acceptable changes between configural, metric and scalar models that demonstrated no significant loss of fit (Chen, 2007). This pattern of results suggests that the DASS-21 items and constructs were operating equivalently across groups and measuring mental health in a consistent manner similar to previous work across other important groupings (e.g., country and gender; Scholten et al., 2017; Lu et al., 2018). This finding is somewhat at odds with suggestions that current measures may be inappropriate for athletes or inapplicable due to the uniqueness of the sport environment (Reardon and Factor, 2010; Chiu et al., 2016). Additionally, and similar to the whole sample, the factor structures of the specific groups (e.g., males and females) provided consistent support for Lovibond and Lovibond’s (1995) model with minimal misspecification observed.

### Convergent Validity

Support for the scales convergent validity was mixed with a range of small to medium effects. Regarding the POMS-D, depression was the only significant predictor, indicating a medium effect. The non-significant beta coefficients for anxiety and stress further demonstrate the convergence of the DASS-21 as a suitable measure of depression. Akin to research outside of sport, the depression subscale positively correlated with other measures of depression supporting its convergent validity (Gloster et al., 2008; Wang et al., 2016). Although frequently utilized in a sport context the POMS-D can also be considered a general measure of depression (Grove and Prapavessis, 1992). The general DASS-21 factor also explained a significant portion of the POMS-D variance.

Next, regarding competitive anxiety, depression, anxiety, and stress all significantly predicted SAS-2 scores. Although, the anxiety subscale was the largest predictor across regression models (e.g., somatic, worry, concentration disruption, and total trait anxiety; Gloster et al., 2008; Wang et al., 2016), depression and stress also positively predicted SAS-2 and subscale scores questioning convergent validity. Note, that a similar pattern was observed for the partial correlations which control for the other predictors supporting the notion that DASS-21 anxiety was the largest predictor of the model. All effects of the DASS-21 on the SAS-2 were small. It is possible that being situated within sport, competitive anxiety is conceptually different to anxiety and can be attributed to other factors (Smith et al., 2006). For example, items of somatic anxiety are similar to items of stress (e.g., “My body feels tense”) and items of worry anxiety are similar to items of depression (e.g., “I have self-doubts”). This finding is nonetheless unsurprising as the DASS-21 was originally conceptualized as a measure of the non-diagnostic general depression-anxiety disorder capturing elements of depression, physical arousal, and psychological tension and agitation (Antony et al., 1998) later separated into depression, anxiety and stress therefore each DASS-21 component is likely to have overlap with other related constructs such as worry and concentration disruption. Likewise, the general DASS-21 factor also explained a significant portion of the SAS-2 total and subscales score variance.

Similarly, regarding the ABQ, stress was the largest predictor of total athlete burnout and exhaustion subscale supporting previous work and convergent validity (De Francisco et al., 2016), whereas depression was the largest predictor of the reduced sense of accomplishment and sport devaluation subscales questioning its convergent validity. Note, that whilst all effects were considered small, all three predictors positively predicted ABQ scores. It is possible that burnout, regardless of context, is the chronic manifestation of stress, and as a result will likely be accompanied by other mental health issues (Raedeke and Smith, 2001; Gustafsson et al., 2018). Nonetheless, research suggests that athlete burnout and mental health (e.g., depression and anxiety) are highly related. For example, depression can be a common outcome of athlete burnout (De Francisco et al., 2016). To date, the DASS-21 stress subscale has received less attention in estimates of convergent validity with research focusing on the depression and anxiety subscales (Gloster et al., 2008; Wang et al., 2016). Therefore, it is likely that the DASS-21 subscales will converge with all aspects of athlete burnout. The significant relationship between the DASS-21 subscales and general measures of stress is in line with previous work (Andreou et al., 2011). The general DASS-21 factor also explained a significant portion of the ABQ total and subscales score variance supporting lack of distinctiveness of the stress subscale.

Finally, regarding athlete psychological strain, depression, anxiety and stress all positively predicted APSQ scores. All effects were considered small. Depression was the largest predictor of total athlete psychological strain and self-regulation subscale, anxiety was the largest predictor of the performance subscale, and depression and anxiety both equally predicted the external coping subscale. The similarly between the DASS-21 and APSQ items may explain these findings. For example, the self-regulation subscale contains items (e.g., “I was less motivated”) which are similar to that of the depression subscale (e.g., “I was unable to become enthusiastic about anything”). Rice et al. (2019) claimed that psychological strain was synonymous with mental health disorders thus supporting the DASS-21’s convergent validity. That is, psychological strain is characterized by emotional exhaustion and difficulty coping which are often linked with depression and anxiety (Rice et al., 2019). The current data is the first study to assess the psychometrics of the APSQ since its development providing evidence of convergent validity and internal consistency. Also, the general DASS-21 factor explained a significant portion of the APSQ total and subscales score variance.

### Limitations and Future Research

Despite several strengths (e.g., two-stage psychometric evaluation in two athlete samples) the present study is not without limitation. The, cut-offs adopted for the ESEM fit indices were recommended for CFA procedures with no ESEM specific indicators developed. Second, ESEM does not enable researchers to test for modification indices or other forms of guided parameter restraint which may reveal further distortions in the data (Marsh et al., 2011). Third, the cross-sectional design and self-report nature of the DASS-21 may confound the data. For example, the DASS-21 offers only a snapshot of recent mental health symptomology and efforts should be made to examine the scales temporal invariance over a playing season. Also, athletes may not wish to disclose mental health symptoms in an attempt to avoid stigma and biases associated with mental health disorders thus subject to social-desirability (Rice et al., 2016).

It is for researchers to address these limitations in future work. Research may wish to refine items for athletic populations by modifying the number of items (i.e., scale purification). Existing research has successfully shortened the DASS-21 creating twelve and nine-item versions of the scale (Kyriazos et al., 2018). Reducing the number of items would provide practical advantages (e.g., participant time commitment) and we believe the current work provides foundation for future work to begin the purification process of the scale for use with athletes. Future work may also wish to contextualize the DASS-21 items for athletic samples and examine whether a domain-specific operationalisation provides greater explanatory value over a non-specific scale (Reardon and Factor, 2010). Moreover, applied researchers have started to examine the effects of career transition (i.e., professional to non-professional) on mental health. For example, Norouzi et al. (2020) claim that retired athletes may be at risk of mental health problems. Norouzi et al. (2020) also reported that a mindfulness-based stress reduction program was able to reduce stress, anxiety and depression and improve psychology well-being in retired Iranian soccer players. Indeed, O’Connor et al. (2020) note that the impact of COVID-19 may extend to economics and professional athletes may not be immune to this (e.g., reduced income from canceled competitions). Thus, future research should extend the current findings to these groups and replication of Norouzi et al. (2020) with the DASS-21 may offer a natural starting point.

Our findings have important implications for research using the DASS-21. For example, support of the scales invariance across gender, athletic expertise, type of sport, and injury status mean that researchers can use the scale to explore several important topics around mental health such as the effect of injury, loss of identity, or reduction in participation as a result of the government lockdowns to minimize transition of COVID-19 (Foskett and Longstaff, 2018; O’Connor et al., 2020). Finally, we suggest that future work investigating the implications of COVID-19 for athletes utilize both general and subscale scores of the DASS-21.

## Conclusion

In conclusion, the psychometrics of the DASS-21 were supported, and researchers can utilize the scale to investigate the implications of COVID-19 in sport. The present findings provide a substantial addition to the athlete mental health literature and support definitive comparisons with the general population, given that the scale operates equivalently across athletes and non-athletes. Moreover, the findings suggest that the DASS-21 relates to other sport-specific measures of mental health such as burnout, psychological strain, anxiety and depression further supports its utility in the sport context. Whilst future research may contextualize or purify the scale for use with athletes, we encourage use of the scale and second recent consensus statements calling for more work exploring athlete’s mental health (Moesch et al., 2018; Henriksen et al., 2019). These findings are useful for those examining the implications of COVID-19 by providing evidence of accuracy and stability of the DASS-21 and thus ensuring rigorous research standards for the field moving forward (O’Connor et al., 2020).

## Data Availability Statement

The raw data supporting the conclusions of this article will be made available by the authors, without undue reservation.

## Ethics Statement

The studies involving human participants were reviewed and approved by York St John University, School of Education, Language, and Psychology Ethics Committee. The patients/participants provided their written informed consent to participate in this study.

## Author Contributions

RV: conceptualization, methodology, formal analysis, investigation, resources, data curation, and writing – original draft, review, and editing. EE: data curation and writing – original draft, review, and editing. TM: writing – original draft, review, and editing. All authors contributed to the article and approved the submitted version.

## Conflict of Interest

The authors declare that the research was conducted in the absence of any commercial or financial relationships that could be construed as a potential conflict of interest.
