# Supplementary material for: Mental Health Measurement in a Post Covid-19 World: Psychometric Properties and Invariance of the DASS-21 in Athletes and Non-athletes
Source: Front Psychol. 2020 Oct 22;11:590559. doi: 10.3389/fpsyg.2020.590559 (PMC7641904; doi:10.3389/fpsyg.2020.590559)
Supplement: Supplementary file 1 [file Table_1.DOCX]

**Supplementary Material**

**Table 4.**

Parameter estimates and latent factor correlations for bifactor ESEM gender invariance DASS-21 models

| Item | Male | | | | | Female | | | | |
| --- | --- | --- | --- | --- | --- | --- | --- | --- | --- | --- |
|  | General | Depression | Anxiety | Stress | r^2^ | General | Depression | Anxiety | Stress | r^2^ |
| Depression |  |  |  |  |  |  |  |  |  |  |
| DASS-21 3 | .602** | **.621**** | .222* | .123 | .555** | .567** | **.688**** | .248* | .061 | .581** |
| DASS-21 5 | **.751**** | .551** | .103 | .019 | 583** | **.787**** | .594** | .136 | .185 | 612** |
| DASS-21 10 | **.753**** | .543** | .112 | .111 | .591** | **.756**** | .586** | .141 | .098 | .633** |
| DASS-21 13 | **.562**** | .486** | .243* | .026 | 487** | **.584**** | .511** | .269* | .163 | 498** |
| DASS-21 16 | .384** | **.718**** | .114 | .102 | .299** | .419** | **.769**** | .154 | .138 | .316** |
| DASS-21 17 | **.804**** | .556** | .237* | .124 | .613** | **.882**** | .599** | .238* | .201 | .638** |
| DASS-21 21 | **.489**** | .411** | .118 | .015 | .525** | **.518**** | .452** | .173 | .072 | .547** |
| Anxiety |  |  |  |  |  |  |  |  |  |  |
| DASS-21 2 | .374** | .133 | **.756**** | .176 | .312** | .408** | .156 | **.811**** | .198 | .328** |
| DASS-21 4 | **.541**** | .214* | .434** | .068 | .477** | **.563**** | .274* | .473** | .062 | .491** |
| DASS-21 7 | **.802**** | .115 | .451** | .087 | .642** | **.847**** | .183 | .489** | .147 | .656** |
| DASS-21 9 | **.763**** | .219* | .473** | .113 | .582** | **.805**** | .264* | .514** | .063 | .596** |
| DASS-21 15 | **.617**** | .133 | .552** | .045 | .516** | **.646**** | .191 | .592** | .201 | .524** |
| DASS-21 19 | **.711**** | .231* | .436** | .166 | .603** | **.747**** | .287* | .497** | .206 | .619** |
| DASS-21 20 | .553** | .117 | **.602**** | .121 | .451** | .609** | .158 | **.658**** | .165 | .467** |
| Stress |  |  |  |  |  |  |  |  |  |  |
| DASS-21 1 | **.632**** | .136 | .054 | .489** | 512** | **.662**** | .162 | .179 | .521** | 531** |
| DASS-21 6 | **.811**** | .111 | .251* | .461** | 651** | **.864**** | .134 | .256* | .506** | 674** |
| DASS-21 8 | **.605**** | .107 | .018 | .473** | .487** | **.649**** | .139 | .057 | .509** | .499** |
| DASS-21 11 | **.703**** | .222* | .102 | .471** | .573** | **.738**** | .274* | .141 | .511** | .596** |
| DASS-21 12 | .512** | .121 | .113 | **.802**** | .638** | .541** | .153 | .153 | **.843**** | .652** |
| DASS-21 14 | .394** | .149 | .241* | **.816**** | .647** | .425** | .184 | .268* | **.836**** | .666** |
| DASS-21 18 | **.741**** | .091 | .035 | .435** | .597** | **.787**** | .139 | .098 | .488** | .611** |
| Correlations |  |  |  |  |  |  |  |  |  |  |
| General |  | .723** | .715** | .774** |  |  | .764** | .731** | .782** |  |
| Depression |  |  | .506** | .552** |  |  |  | .534** | .562** |  |
| Anxiety |  |  |  | .538** |  |  |  |  | .559** |  |

Note. Values in bold indicate highest loading on that factor. Values underlined indicate intended factor. N = 894. * p < .05; ** p < .01.

**Table 5.**

Parameter Estimates for Bifactor-ESEM Athletic Expertise Invariance DASS-21 Models

| Item | Non-Athletes | | | | | Amateur Athletes | | | | | Elite Athletes | | | | |
| --- | --- | --- | --- | --- | --- | --- | --- | --- | --- | --- | --- | --- | --- | --- | --- |
|  | General | Depression | Anxiety | Stress | r^2^ | General | Depression | Anxiety | Stress | r^2^ | General | Depression | Anxiety | Stress | r^2^ |
| Depression |  |  |  |  |  |  |  |  |  |  |  |  |  |  |  |
| DASS-21 3 | .562** | **.617**** | .221* | .063 | .492** | .584** | **.626**** | .224* | .051 | .504** | .593** | **.643**** | .236* | .064 | .513** |
| DASS-21 5 | **.734**** | .563** | .023 | .137 | .603** | **.752**** | .574** | .017 | .126 | .617** | **.767**** | .598** | .048 | .131 | .624** |
| DASS-21 10 | **.708**** | .541** | .105 | .141 | .588** | **.723**** | .563** | .128 | .164 | .601** | **.736**** | .584** | .133 | .159 | .615** |
| DASS-21 13 | **.503**** | .469** | .229* | .067 | .436** | **.518**** | .482** | .233* | .042 | .445** | **.529**** | .499** | .247* | .103 | .456** |
| DASS-21 16 | .388** | **.722**** | .107 | .145 | .602** | .403** | **.741**** | .122 | .112 | .613** | .433** | **.762**** | .165 | .136 | .627** |
| DASS-21 17 | **.804**** | .556** | .231* | .113 | .694** | **.827**** | .569** | .228* | .146 | .705** | **.814**** | .584** | .249* | .172 | .711** |
| DASS-21 21 | .352** | **.437**** | .082 | .082 | .362** | .365** | **.452**** | .104 | .042 | .378** | .396** | **.466**** | .191 | .069 | .394** |
| Anxiety |  |  |  |  |  |  |  |  |  |  |  |  |  |  |  |
| DASS-21 2 | .364** | .109 | **.763**** | .089 | .655** | .381** | .103 | **.782**** | .074 | .647** | .362** | .234* | **.795**** | .061 | .654** |
| DASS-21 4 | **.528**** | .228* | .427** | .004 | .491** | **.546**** | .226* | .439** | .088 | .488** | **.568**** | .221* | .512** | .121 | .496** |
| DASS-21 7 | 403** | .013 | **.796**** | .057 | .682** | 421** | .051 | **.811**** | .103 | .701** | 413** | .087 | **.803**** | .136 | .713** |
| DASS-21 9 | **.743**** | .247* | .462** | .106 | .698** | **.768**** | .234* | .483** | .123 | .671** | **.777**** | .243* | .497** | .188 | .687** |
| DASS-21 15 | **.599**** | .007 | .561** | .051 | .511** | **.615**** | .148 | .573** | .072 | .526** | **.626**** | .152 | .584** | .002 | .532** |
| DASS-21 19 | .432** | .232* | **.703**** | .123 | .622** | .457** | .248* | **.714**** | .165 | .629** | .469** | .236* | **.732**** | .136 | .634** |
| DASS-21 20 | **.584**** | .118 | .512** | .002 | .541** | **.601**** | .104 | .569** | .031 | .537** | **.638**** | .065 | .582** | .004 | .549** |
| Stress |  |  |  |  |  |  |  |  |  |  |  |  |  |  |  |
| DASS-21 1 | **.613**** | .062 | .153 | .496** | .557** | **.642**** | .044 | .143 | .488** | .545** | **.656**** | .215* | .007 | .488** | .545** |
| DASS-21 6 | .452** | .058 | .252* | **.801**** | .688** | .476** | .108 | .247* | **.816**** | .702** | .481** | .167 | .256* | **.816**** | .702** |
| DASS-21 8 | **.636**** | .131 | .008 | .482** | .632** | **.621**** | .104 | .029 | .474** | .621** | **.635**** | .159 | .024 | .474** | .621** |
| DASS-21 11 | **.657**** | .241* | .102 | .459** | .625** | **.703**** | .233* | .216* | .477** | .612** | **.714**** | .244* | .002 | .477** | .612** |
| DASS-21 12 | **.763**** | .038 | .107 | .476** | .658** | **.814**** | .054 | .114 | .505** | .679** | **.831**** | .187 | .148 | .505** | .679** |
| DASS-21 14 | .412** | .109 | .243* | **.811**** | .662** | .387** | .126 | .252* | **.831**** | .696** | .398** | .163 | .261* | **.831**** | .696** |
| DASS-21 18 | .469** | .081 | .007 | **.722**** | .641** | .445** | .092 | .053 | **.748**** | .653** | .461** | .121 | .134 | **.748**** | .653** |
| Correlations |  |  |  |  |  |  |  |  |  |  |  |  |  |  |  |
| General |  | .688** | .643** | .701** |  |  | .714** | .679** | .739** |  |  |  | .727** | .691** | .752** |
| Depression |  |  | .536** | .574** |  |  |  | .564** | .597** |  |  |  |  | .589** | .608** |
| Anxiety |  |  |  | .561** |  |  |  |  | .588** |  |  |  |  |  | .593** |

Note. Values in bold indicate highest loading on that factor. Values underlined indicate intended factor. N = 894. * p < .05; ** p < .01.

**Table 6.**

Parameter estimates and latent factor correlations for bifactor ESEM sport type invariance DASS-21 models

| Item | Individual | | | | | Team | | | | |
| --- | --- | --- | --- | --- | --- | --- | --- | --- | --- | --- |
|  | General | Depression | Anxiety | Stress | r^2^ | General | Depression | Anxiety | Stress | r^2^ |
| Depression |  |  |  |  |  |  |  |  |  |  |
| DASS-21 3 | .564** | **.601**** | .214* | .184 | .517** | .592** | **.697**** | .277* | .009 | .563** |
| DASS-21 5 | **.717**** | .536** | .006 | .163 | 563** | **.804**** | .614** | .187 | .147 | 628** |
| DASS-21 10 | **.689**** | .527** | .165 | .123 | .571** | **.787**** | .627** | .196 | .164 | .641** |
| DASS-21 13 | **.524**** | .455** | .247* | .005 | 512** | **.599**** | .523** | .282* | .012 | 507** |
| DASS-21 16 | .372** | **.692**** | .102 | .044 | .364** | .446** | **.791**** | .193 | .164 | .333** |
| DASS-21 17 | **.764**** | .535** | .258* | .009 | .588** | **.874**** | .616** | .244* | .002 | .647** |
| DASS-21 21 | **.481**** | .421** | .009 | .191 | .509** | **.569**** | .487** | .002 | .196 | .593** |
| Anxiety |  |  |  |  |  |  |  |  |  |  |
| DASS-21 2 | .363** | .025 | **.744**** | .132 | .306** | .428** | .169 | **.811**** | .129 | .335** |
| DASS-21 4 | **.536**** | .263* | .421** | .098 | .463** | **.584**** | .281* | .473** | .113 | .499** |
| DASS-21 7 | **.788**** | .158 | .436** | .074 | .637** | **.811**** | .194 | .489** | .005 | .662** |
| DASS-21 9 | **.759**** | .228* | .444** | .033 | .565** | **.787**** | .288* | .514** | .007 | .603** |
| DASS-21 15 | **.602**** | .002 | .539** | .189 | .502** | **.668**** | .108 | .592** | .124 | .535** |
| DASS-21 19 | **.703**** | .267* | .422** | .005 | .594** | **.791**** | .236* | .497** | .008 | .622** |
| DASS-21 20 | .533** | .007 | **.587**** | .164 | .439** | .628** | .005 | **.658**** | .198 | .478** |
| Stress |  |  |  |  |  |  |  |  |  |  |
| DASS-21 1 | **.598**** | .002 | .136 | .446** | 501** | **.671**** | .187 | .056 | .534** | 542** |
| DASS-21 6 | **.765**** | .168 | .258* | .443** | 634** | **.832**** | .006 | .288* | .528** | 684** |
| DASS-21 8 | **.584**** | .124 | .107 | .451** | .463** | **.658**** | .166 | .168 | .513** | .506** |
| DASS-21 11 | **.659**** | .263* | .003 | .432** | .547** | **.746**** | .294* | .082 | .537** | .603** |
| DASS-21 12 | .488** | .147 | .134 | **.761**** | .601** | .558** | .128 | .134 | **.812**** | .668** |
| DASS-21 14 | .412** | .009 | .252* | **.778**** | .613** | .431** | .068 | .282* | **.808**** | .671** |
| DASS-21 18 | **.702**** | .111 | .147 | .421** | .565** | **.795**** | .107 | .143 | .497** | .629** |
| Correlations |  |  |  |  |  |  |  |  |  |  |
| General |  | .711** | .702** | .731** |  |  | .769** | .742** | .758** |  |
| Depression |  |  | .497** | .522** |  |  |  | .552** | .569** |  |
| Anxiety |  |  |  | .514** |  |  |  |  | .578** |  |

Note. Values in bold indicate highest loading on that factor. Values underlined indicate intended factor. N = 894. * p < .05; ** p < .01.

**Table 7.**

Parameter estimates and latent factor correlations for bifactor ESEM injury status invariance DASS-21 models

| Item | Injured | | | | | Non-Injured | | | | |
| --- | --- | --- | --- | --- | --- | --- | --- | --- | --- | --- |
|  | General | Depression | Anxiety | Stress | r^2^ | General | Depression | Anxiety | Stress | r^2^ |
| Depression |  |  |  |  |  |  |  |  |  |  |
| DASS-21 3 | .564** | **.601**** | .233* | .074 | .513** | .592** | **.697**** | .217* | .058 | .562** |
| DASS-21 5 | **.701**** | .531** | .147 | .138 | 547** | **.767**** | .604** | .108 | .136 | 628** |
| DASS-21 10 | **.712**** | .514** | .136 | .162 | .553** | **.798**** | .609** | .116 | .108 | .647** |
| DASS-21 13 | .443** | **.497**** | .256* | .067 | 442** | .552** | **.596**** | .251* | .027 | 506** |
| DASS-21 16 | .364** | **.689**** | .128 | .178 | .282** | .439** | **.781**** | .132 | .104 | .334** |
| DASS-21 17 | **.762**** | .525** | .247* | .081 | .591** | **.817**** | .632** | .221* | .058 | .642** |
| DASS-21 21 | **.449**** | .388** | .262* | .007 | .503** | **.562**** | .487** | .207* | .016 | .553** |
| Anxiety |  |  |  |  |  |  |  |  |  |  |
| DASS-21 2 | .371** | .228* | **.748**** | .006 | .316** | .413** | .262* | **.816**** | .005 | .347** |
| DASS-21 4 | .422** | .231* | **.509**** | .061 | .464** | .482** | .287* | **.574**** | .062 | .507** |
| DASS-21 7 | **.788**** | .005 | .448** | .074 | .623** | **.836**** | .192 | .498** | .161 | .669** |
| DASS-21 9 | **.759**** | .236* | .467** | .124 | .571** | **.812**** | .271* | .523** | .033 | .604** |
| DASS-21 15 | **.603**** | .024 | .548** | .165 | .503** | **.661**** | .102 | .605** | .191 | .533** |
| DASS-21 19 | **.688**** | .282* | .426** | .107 | .589** | **.751**** | .282* | .507** | .204 | .627** |
| DASS-21 20 | .541** | .185 | **.592**** | .008 | .436** | .623** | .002 | **.663**** | .184 | .481** |
| Stress |  |  |  |  |  |  |  |  |  |  |
| DASS-21 1 | **.588**** | .181 | .167 | .463** | 501** | **.658**** | .102 | .132 | .533** | 539** |
| DASS-21 6 | **.758**** | .132 | .262* | .434** | 633** | **.826**** | .121 | .262* | .512** | 677** |
| DASS-21 8 | .447** | .222* | .104 | **.582**** | .465** | .512** | .242* | .161 | **.658**** | .504** |
| DASS-21 11 | **.676**** | .244* | .057 | .456** | .552** | **.742**** | .281* | .167 | .536** | .603** |
| DASS-21 12 | .487** | .134 | .193 | **.732**** | .611** | .563** | .003 | .159 | **.831**** | .667** |
| DASS-21 14 | .365** | .052 | .253* | **.751**** | .626** | .438** | .113 | .274* | **.822**** | .674** |
| DASS-21 18 | **.707**** | .261* | .082 | .422** | .574** | **.791**** | .175 | .181 | .496** | .623** |
| Correlations |  |  |  |  |  |  |  |  |  |  |
| General |  | .711** | .687** | .712** |  |  | .771** | .734** | .777** |  |
| Depression |  |  | .521** | .567** |  |  |  | .545** | .576** |  |
| Anxiety |  |  |  | .531** |  |  |  |  | .563** |  |

Note. Values in bold indicate highest loading on that factor. Values underlined indicate intended factor. N = 894. * p < .05; ** p < .01.
